# Supplementary material for: Chlorogenic Acid of Cirsium japonicum Resists Oxidative Stress Caused by Aging and Prolongs Healthspan via SKN-1/Nrf2 and DAF-16/FOXO in Caenorhabditis elegans
Source: Metabolites. 2023 Feb 3;13(2):224. doi: 10.3390/metabo13020224 (PMC9959019; doi:10.3390/metabo13020224)
Supplement: Supplementary file 1 [file metabolites-13-00224-s001.zip › metabolites-2116568-supplementary.pdf]

Table S1. High performance liquid chromatography (HPLC) analysis condition of phenolic compounds from CJ.

HPLC (UltiMate 3000, Thermo Scientific, CA, USA)	
Column	Waters symmetry C18 column (Waters, 4.6 × 150 mm, 5 μm)
Solvent	(A) acetonitrile<br>(B) 0.02% (v/v) aqueous phosphoric acid<br>Gradient: 87% solvent B for 6 min, 85–87% solvent B for the next 3 min, 81–85% solvent B for 17 min, 72–81% solvent B for 28 min and a linear step from 72 to 87% solvent B for 12 min.
Column temperature	35℃
Wavelength	340 nm
Flow rate	1.0 mL/min

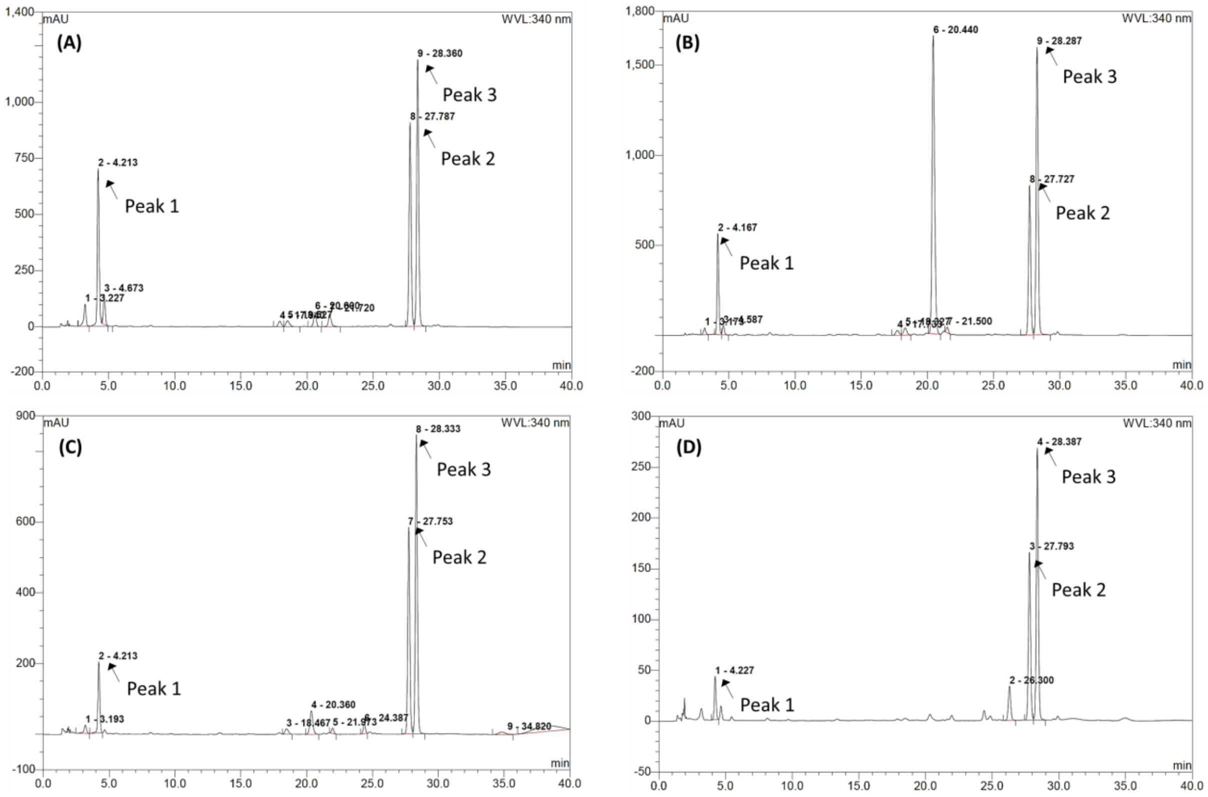

Figure S1. High performance chromatography profiles of phenolics in CJ collected from four different regions in Korea. (A) Pocheon, (B) Goheung, (C) Jeju, and (D) Yesan. peak 1: chlorogenic acid, peak 2: linarin and peak 3: pectolinarin.

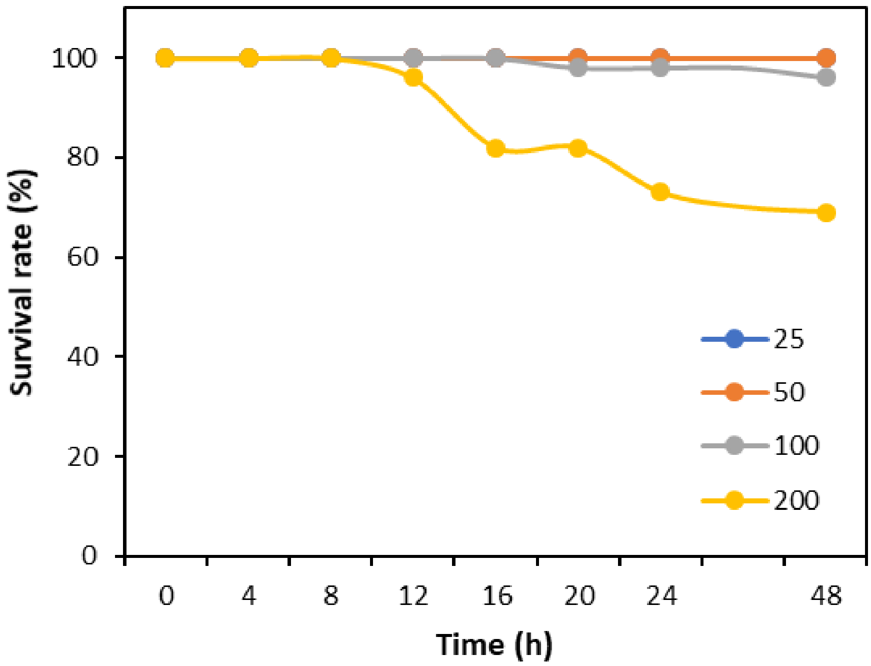

Figure S2. Acute toxicity by the concentrations of chlorogenic acid (CA). *C. elegans* was treated with CA at a concentration of 25-200 μg/mL.
